# Firearm Injuries Presenting to a Tertiary Care Hospital of Karachi, Pakistan

**DOI:** 10.5249/jivr.v1i1.27

**Published:** 2009-07

**Authors:** Muazzam Nasrullah, Junaid A Razzak

**Affiliations:** ^*a*^Department of Emergency Medicine, The Aga Khan University, Stadium Road PO Box 3500, Karachi 74800, Pakistan; ^*b*^Injury Control Research Center, West Virginia University, PO Box 9151, Morgantown, West Virginia 26506, USA; ^*c*^Department of Community Medicine, West Virginia University School of Medicine, Health Science Center, Morgantown, WV 26506, USA

## Abstract

**Background:**

Violence is a public health problem in low and middle income countries. Our study attempted to define the circumstances, risk groups, extent and severity of firearm-related injuries in patients coming to the Aga Khan University Hospital (AKUH) Karachi, Pakistan.

**Methods:**

This was a retrospective study conducted in the department of Emergency Medicine (EM) at AKUH Karachi, Pakistan. Past medical records of all patients who were injured by firearms and were presented to the AKUH Emergency Department (ED) from June 2002 till May 2007 were reviewed. Data were recorded on the basic demographics of injured, length of hospital stay, body parts injured and the outcome (alive vs. dead).

**Results:**

Total of 286 patients with firearm injuries were identified. Majority of them were males (92%; n=264). More than half of the patients (63%) were in the age group of 21-40 years. Upon arrival to the hospital 85% (n=243) of patients had Glasgow Coma Scale (GCS)>= 13. The mean injury severity score (ISS) was found to be 6 (SD ±4). The length of hospital stay of patients ranged from 0 to 54 days with a mean of 7 days. Lower limb were the most affected body parts (30%, n=86) followed by abdomen pelvis (27%, n=77). Seven percent (n=21) of the patient who were brought to the hospital were labeled as "deceased on arrival". Most of the injuries were caused during the act of robbery (40%, n=103) in the city.

**Conclusions:**

Robbery was the most common cause of firearm injuries. Lower limb, abdomen and pelvis were the most affected body regions. Educational efforts, and individual, community and societal approaches are needed to alleviate firearm-related injuries.

## Introduction

Violent injuries are the eighth leading cause of death, worldwide.^1^ Besides high death toll firearm injuries cause significant morbidity, long-term physical and psychological disability for individuals, families, communities and societies.^2^ In 1996, the World Health Assembly declared violence as a leading global public health problem high-lighting a need for implementing the global strategy to prevent it.^3^ Firearms and their use are modifiable risk factors,^4^ which if recognized and addressed, could help decrease the burden of violent deaths.

Violent injuries are common in the low and middle income countries. In 2000, the rate of violence-related death in low-to middle-income countries as a whole was more than twice that in high-income countries, although rates vary between regions and within countries.^5^

Picture of the violent deaths in Pakistan is not different from other low income countries. Early studies have shown that firearms were a common mode of violent deaths among young males in Karachi, Pakistan^6^ with death rate being 4.22/100,000 per year.^7^ Both high velocity rifles (Kalashnikov, Rifles, Pistols), and low velocity weapons and shotguns were used for firearm injuries.^8^

Our study attempted to define the circumstances, risk groups, extent and severity of firearm-related injuries in patients coming to the tertiary care hospital of Karachi, Pakistan.

## Methods

**a. Study design and Setting**

This is a retrospective study conduced at the Aga Khan University Hospital (AKUH), Karachi Pakistan. The AKUH is a private 500-bedded, fee-for-service, urban tertiary care teaching hospital located in Karachi. Karachi, a city of fourteen million people is the largest and most populous city of Pakistan accounting for 10 % of total population and 30% of the urban population of Pakistan. It is the hub of economic activity in Pakistan.

The Emergency Department (ED) at AKUH has an annual census of approximately 47, 000 patients including both adults and pediatrics. The hospital is not a designated government trauma centre but still receives trauma patients who are usually referred from other hospitals. Most patients use private means of transportation to come to the AKUH-ED due to the of lack of established pre-hospital care infrastructure.^9^

**b. Data Collection and Subjects**

Log books containing the information of all patients presented to the AKUH-ED were accessed. The injured of all ages and gender who came to the ED because of firearm related injuries during June 2002-May 2007 were selected. The patients with intentional and non-intentional injuries were included. A specific data collection form was developed to extract data from medical records of selected patients on basic demographics of injured, date and time of arrival, length of hospital stay, referral to and from hospital, circumstances surrounding injuries and eventual outcome (deaths vs. discharge from hospital). Medical records of the patients were reviewed and data were documented. Glasgow Comma Scale (GCS) which recorded at the time of patients’ presentation to the hospital were also noted. Severity of injury was measured by the Injury Severity Score (ISS).^10^ Injured body regions were defined as those associated with the computation of the ISS: head and neck, face, thorax, abdomen, extremities, and skin. The additional category of “multiple body regions” indicates injuries to > 1 of the above regions.

**c. Data Analysis**

All the data were manually checked, coded and entered into a database then analyzed using SPSS version 15 software. The descriptive statistics for demographics of injured, length of hospital stay, body parts injured and the outcome were calculated. Odds ratios (ORs) with 95% confidence interval (CI) among males and females were also calculated.

## Results

A total of 286 patients presented to AKUH with firearm injuries during five years. Males outnumbered female by 12:1 accounting for 92% (n=264) of injured. More than half of the patients (63%) were in the age group of 21-40 years followed by 40-60 years (18%), 1-20 years (17%) and ≥60 years (2%).

Upon arrival to hospital 85% (n=243) patients had Glas-gow Comma Scale (GCS) ≥ 13 (minor) while 6% (n=17) had GCS ≤ 8 (severe) and only 2% (n=5) had GCS 9-12 (moderate). The hospital length of stay for patients ranged from 0 to 54 days with a mean of 7. The mean injury severity score (ISS) was found to be 6 (SD ±4).

Lower limbs were the most affected body parts (30%, n=86) followed by abdomen pelvis (27%, n=77), head and neck 23% (n=65), upper limbs 21% (n=61) and Thorax 18% (n=52). Multiple injuries (more than one body part effected) were reported in 21% (n=61) of cases. Seven percent (n=21) of the patients were labeled as ‘deceased on arrival’ to the hospital.

Robbery was found to be the most common reason for the firearm use (40%, n=103). Bystanders 19% (n=49), accidental gunshots (10%, n=25), quarrel (6%, n=16), police encounter 4% (n=10) and self harm 1% (n=3), were the other reasons for which firearms were used. However, 12.5% (n=32) and 3.1% (n=8) of cases were grouped into the “others” (i.e., cases which did not fit into any of the above category) and “unknown” categories, respectively. Majority of the patients were presented in ED between 21-24 hours (23%) followed by 17-20 hours (22%) and 0-4 hours (22%) respectively.

None of the odds between males and females were found to be statistically significant. The distribution of firearm injuries by gender is shown in Table 1.

**Table:** Table 1: **Distribution of Firearm injuries by Gender**

Variables	Male N (%)	Females N (%)	Total N (%)	Odds Ratios (95% CI)
Age Groups				
0-10	6 (2.3)	3 (13.6)	9 (3.1)	0.2 (0.0-0.6)*
11-20	38 (14.4)	2 (9.1)	40 (14.0)	1.7 (0.4-7.5)
21-30	89 (33.7)	5 (22.7)	94 (32.9)	1.7 (0.6-4.8)
31-40	83 (31.4)	3 (13.6)	86 (30.1)	2.9 (0.8-10.1)
41-50	34 (12.9)	3 (13.6)	37 (12.9)	0.9 (0.3-3.3)
51-60	11 (4.2)	3 (13.6)	14 (4.9)	0.3 (0.1-1.1)
61-70	3 (1.1)	1 (4.5)	4 (1.4)	0.2 (0.0-2.4)
70+	0 (0)	2 (9.1)	2 (0.7)	-
GCS				
Severe <8	17 (6.4)	0 (0)	17 (6.4)	-
Moderate 9-12	5 (1.9)	0 (0)	5 (1.9)	-
Minor >13	225 (85.2)	18 (100.0)	243 (91.7)	1.2 (0.4-4.0)
ISS†				
1-9	221 (89.8)	19 (95.0)	240 (90.2)	0.8 (0.2-2.9)
10-19	20 (8.1)	1 (5.0)	21 (7.9)	1.7 (0.2-13.5)
20-75	5 (2.0)	0 (0)	5 (1.9)	-
Body Parts Injured				
Lower Limb	79 (31.6)	7 (35.0)	86 (31.9)	0.9 (0.4-2.3)
Abdomen & Pelvis	72 (28.8)	5 (25.0)	61(22.6)	1.3 (0.5-3.6)
Head & Neck	61 (24.4)	4 (20.0)	65 (24.1)	1.4 (0.4-4.1)
Upper Limb	54 (21.6)	7 (35.0)	77 (28.5)	0.6 (0.2-1.4)
Thorax	49 (19.6)	3 (15.0)	52 (19.3)	1.4 (0.4-5.1)
Multiple Injuries	56 (22.5)	5 (25.0)	61 (22.7)	0.9 (0.3-2.6)
Causes of Firearm Use				
Robbery	93 (39.9)	10 (45.5)	103 (40.4)	0.7 (0.3-1.6)
Accidental	22 (9.4)	3 (13.6)	25 (9.8)	0.6 (0.2-2.1)
Quarrel	15 (6.4)	1 (4.5)	16 (6.3)	1.3 (0.2-10.1)
Dead	8 (3.4)	1 (4.5)	9 (3.5)	0.7 (0.1-5.5)
Police encounter	10 (4.3)	0(0)	10 (3.9)	-
Self	3 (1.3)	0(0)	3 (1.2)	-
Bystanders	46 (19.7)	3 (13.6)	49 (19.2)	1.3 (0.4-4.7)
Others ††	28 (12.0)	4 (18.2)	32 (12.5)	0.5 (0.2-1.7)
Unknown	8 (3.4)	0 (0)	8 (3.1)	-

* p< 0.05 † ISS: Injury Severity Score †† Others: Include cases which do not fall into any of the category “Causes of Firearm Use”

## Discussion

Our study showed that young males were at higher risk for firearm related injuries than females. Lower limbs, and abdomen and pelvis were the major body regions affected. Majority of the patients were found to have GCS ≥ 13 on arrival to the hospital and mean ISS was 6. Patient stayed on average seven days in hospital. Robbery was found to be the main cause of firearm injury.

Male gender has been described as high risk group in other studies ^4,6,7^ and age group of 21-40 years was found to be the most affected group, as depicted in studies from Pakistan.^11,12^ However incidence of firearm related injuries among females in Pakistan in also reported.^13^ Our finding of high proportion of firearm related injuries among male could be due to their gender role which obligate them to be more exposed to the outside environment than females. The lower limb, and abdomen and pelvis were the primary areas of body where most of injuries occurred. These findings are different than other studies where head and chest were the major body parts affected.^11,14^ Patients with head injury are most likely to die before getting to hospital thus in this study we could not capture injury related characteristics of this population. Another reason could be the robber’s intention in disabling victims rather than killing. Most of the injuries in our study were caused during the act of robbery followed by accidental gunshots and quarrel, though there is evidence that a minority of home invasion crimes resulted in injury.^15^ The information on whether injured were victims or offenders during the act of robbery was missing on medical records thus making it difficult to prioritize the prevention efforts, more research is needed to address this issue.

Proportion (11%) of injuries in our study was accidental or suicidal. Studies have shown that firearms are frequently used as a mode of suicidal deaths^16,17^  while accidental deaths are also not uncommon.^18^ Mostly accidental deaths occurred while mishandling the firearms,^19^ gun cleaning and behavior to impress others.^20^ Educational efforts and inaccessibility of guns to high risk groups are important steps in preventing firearm related incidents.

The severity of injuries was assessed by GCS, ISS and length of hospital stay which were on lower side. These findings are in line with the study,^21^ however the conclusions can be drawn with caution as the severity of wounds caused by a firearms depends mainly on the distance from the shooter and whether plastic pellet has ended up in the wound incurred at a short distance.^22^ AKUH is a tertiary care hospital in private setting. In many of the cases this hospital is a secondary site of referral from other government hospitals thus making it likely to present the patients with less severity and so is the mortality.

The epidemiology of firearm injuries requires a range of strategies for prevention. These strategies should use ecological model to help them to get to the root of violence.^5^ Individual approaches include social development programs in particular to help young people to develop social skills, manage anger and resolve conflicts. Community based efforts comprise of modification of physical environment, such as improving street lighting, creating safe routes for youth on their way to and from work places. In addition, community policing and coordinated community interventions aiming toward improving services are some of the useful measures for firearm crime reduction. Societal approaches aiming for legislative and judicial remedies, proper licensing of firearm use and policy changes to reduce poverty and inequality are important measures in reducing firearm injuries in the city of Karachi.

This is a single centre study thus difficult to generalize the results and to calculate the rate of firearm injuries in our population. As AKUH being the referral centre there is a possibility of selection bias of patients in terms of its severity. Other types of violent injuries like bomb blasts and burns are not included in our study though they are common in the city.^23^ Despite these limitations, our paper provides valuable information on firearm-related injuries in a low-income setting. In addition, it provides some of the approaches to prevent these kinds of injuries.

## Conclusion

Firearm injuries were common among young males in our hospital. Robbery was the most common cause of firearm injuries. Lower limb and abdomen and pelvis were the most affected body regions. Educational efforts, and individual, community and societal approaches are needed to alleviate firearm-related injuries.
